# Perturbation of Host Nuclear Membrane Component RanBP2 Impairs the Nuclear Import of Human Immunodeficiency Virus -1 Preintegration Complex (DNA)

**DOI:** 10.1371/journal.pone.0015620

**Published:** 2010-12-14

**Authors:** Ruonan Zhang, Rajeev Mehla, Ashok Chauhan

**Affiliations:** Department of Pathology, Microbiology and Immunology, University of South Carolina School of Medicine, Columbia, South Carolina, United States of America; Johns Hopkins School of Medicine, United States of America

## Abstract

HIV-1 is a RNA virus that requires an intermediate DNA phase via reverse transcription (RT) step in order to establish productive infection in the host cell. The nascent viral DNA synthesized via RT step and the preformed viral proteins are assembled into pre-integration complex (PIC) in the cell cytoplasm. To integrate the viral DNA into the host genome, the PIC must cross cell nuclear membrane through the nuclear pore complex (NPC). RanBP2, also known as Nup358, is a major component of the cytoplasmic filaments that emanates from the nuclear pore complex and has been implicated in various nucleo-cytoplasmic transport pathways including those for HIV Rev-protein. We sought to investigate the role of RanBP2 in HIV-1 replication. In our investigations, we found that RanBP2 depletion via RNAi resulted in profound inhibition of HIV-1 infection and played a pivotal role in the nuclear entry of HIV DNA. More precisely, there was a profound decline in 2-LTR DNA copies (marker for nuclear entry of HIV DNA) and an unchanged level of viral reverse transcription in RanBP2-ablated HIV-infected cells compared to RanBP3-depleted or non-specific siRNA controls. We further demonstrated that the function of Rev was unaffected in RanBP2-depleted latently HIV infected cells (reactivated). We also serendipitously found that RanBP2 depletion inhibited the global ectopic gene expression. In conclusion, RanBP2 is a host factor that is involved in the nuclear import of HIV-1 PIC (DNA), but is not critical to the nuclear export of the viral mRNAs or nucleo-cytoplasmic shuttling of Rev. RanBP2 could be a potential target for efficient inhibition of HIV.

## Introduction

The nuclear pore complexes (NPCs) are channels in the nuclear membrane that regulate trafficking across the nuclear envelope (NE) [1]. NPCs are composed of multiple copies of approximately 30 proteins, known as nucleoporins (Nups) [2]–[5]. Most of the Nups contain phenylalanine-glycine (FG) repeats, which line the central channel and extend on both the cytoplasmic and the nucleoplasmic faces. The FG repeat domains are responsible for interaction with transport receptors and thus provide binding sites for receptor-cargo complexes to allow selective entry across the NPCs [6]–[9]. RanBP2, also known as Nup358, is a major component of cytoplasmic filaments of NPC [10]–[12].

Several studies showed that RanBP2 regulates the shuttling of HIV-1 Rev protein between the cytoplasm and the nucleus [13]–[14]. Rev protein promotes the nuclear export of Rev responsive element (RRE)-containing unspliced or partially spliced HIV mRNA species [15]–[16]. HIV-Rev bears a nuclear localization signal (NLS) and a leucine-rich nuclear export signal (NES) that allow it to continuously shuttle between the cytoplasm and the nucleus [17]–[18]. Upon Rev synthesis in the cytoplasm, the import receptors bind to its NLS, leading to its transportation into the nucleus [18]. Once in the nucleus, Rev binds with RRE of HIV-mRNAs and the leucine-rich NES of Rev is recognized by CRM1, a nuclear export receptor that facilitates the nuclear export of mRNA-Rev complex [17], [19], [20]. It is shown that depletion of RanBP2 by siRNA strongly inhibits the nuclear import of HIV-1 Rev protein [13]. Subsequently, another study showed that RanBP2 depletion blocked CRM1-mediated NES protein export and caused predominant nuclear accumulation of HIV-1 Rev protein [14]. Since shuttling of HIV-1 Rev is required for the nuclear export of HIV mRNAs and for the virus to complete its life cycle, the role of RanBP2 becomes essential for HIV-1 replication.

The capacity of HIV-1 to infect both the dividing and non-dividing cells indicates that the viral DNA assembles into HIV-1 preintegration complex (PIC) and actively crosses the nuclear envelope by taking advantage of the cellular nuclear import machinery. The nuclear import of HIV-1 DNA is a critical step in viral life cycle and the mechanism for nuclear entry is still poorly understood. Upon HIV-1 infection, the genomic viral RNA is reverse transcribed into viral DNA. The nascent viral DNA assembles into PIC with a three-stranded DNA, specified by the central polypurine tract central sequence (cPPT-CTS), and several viral proteins such as nucleocapsid (NC), matrix protein (MA), reverse transcriptase (RT), integrase (IN) and Vpr proteins [21]–[28]. Some of these proteins in the PIC carry karyophilic signals and thus can be recognized by the nuclear import receptors that allow subsequent translocation of the whole complex into the nucleus via cellular active transport machinery [21], [29]–[30]. Albeit, the role of IN, MA, Vpr and cPPT-CTS in the nuclear import of HIV DNA has been refuted by several studies [31]–[39], [69], however, a recent study has shown a proven role for cPPT-CTS in the PIC nuclear import [28]. Yet, more studies are needed to validate specifically which viral components are required for the PIC import.

In addition, to viral components the NPC components, obviously, are required in the process to deliver the PIC into the nucleus. The PIC cargo is transported across the NPC involving the network of several Nups and import receptors [40]. It has been shown that Nup153, a major component of nuclear filament of NPC, directly interacts with the viral IN, a component of HIV-1 PIC, and is essential for the nuclear entry of HIV-1 DNA [41]. Also, depletion of Nup98, which is located near the nuclear basket and dynamically associates with and dissociates from the nuclear pore, impairs the nuclear import of HIV-1 cDNA [42]. In the two genome wide siRNA screening studies [40], [43] and a functional study [44] show that TNPO3 or transportin 3, a member of karyopherin beta protein family, is intimately involved in the PIC nuclear import. This was further validated by another study wherein the viral capsid (CA) protein instead of IN is a major viral protein that mediates the transportation of PIC into the nucleus [45]. Interestingly, in global siRNA screening study, Nup153, Nup214 (a major component of distal ring) and RanBP2/Nup358 were identified to be essential nucleoporins responsible for mediating the nuclear import of HIV-1 DNA [40]. Thus, it is possible that RanBP2 is a part of the Nups network and plays an essential role in translocating HIV-1 PIC from the cytoplasm into the nucleus.

Based on the earlier evidences that RanBP2 is involved in regulation of HIV-1 Rev nucleo-cytoplasmic trafficking and HIV DNA import, we sought to investigate the role of RanBP2 in HIV-1 replication using RNAi and HIV infection. We used RNAi strategy to deplete RanBP2 and found that the nuclear entry of HIV-1 DNA was largely impaired, leading to profound inhibition of HIV-1 replication. We also found that RanBP2 ablation had no effect on the viral mRNA export. Further, we serendipitously found that RanBP2 depletion universally inhibited the ectopic gene expression and might not be necessarily specific for the HIV.

## Results

### RNAi mediated knockdown of RanBP2 and RanBP3

**Figure 1:**
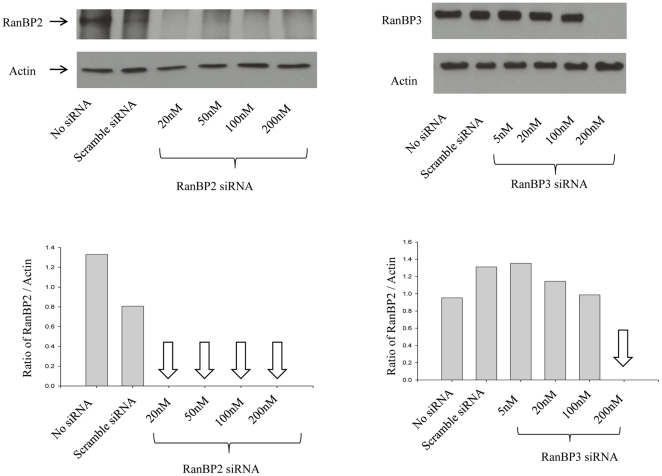
siRNA-mediated depletion of RanBP2 and RanBP3. SVGA cells were transfected either with RanBP2 siRNA or RanBP3 siRNA, respectively. In parallel, control SVGA cells were either transfected with 200 nM scrambled siRNA or without siRNA. Total proteins were extracted 72 h post siRNA transfection. Western blot analysis was performed to examine levels of RanBP2 and RanBP3, respectively. Actin was used as internal control. Image J was used to analyze the intensities of the bands.

We sought to examine the role of RanBP2 in HIV-1 life cycle, in particular the possible role in Rev-function and PIC nuclear import. In order to investigate the role of RanBP2, RNAi approach was validated using specific siRNA. Western blot analysis showed that when specific siRNA was transfected at concentration as low as 20 nM, RanBP2 protein was robustly knocked down after 72 hrs (**Fig. 1**). The scrambled siRNA and the cells without siRNA were used as controls. As an internal control to exclude the unexpected non-specific siRNA effects in the functional assays we also included siRNA targeting Ran-binding protein 3 (RanBP3), an accessory factor in Ran GTPase system [53]. Similar to RanBP2 siRNA, RanBP3 siRNA ablated RanBP3 protein completely when used at 200 nM concentrations (**Fig. 1**).

### RanBP2 regulates ectopic gene expression

**Figure 2:**
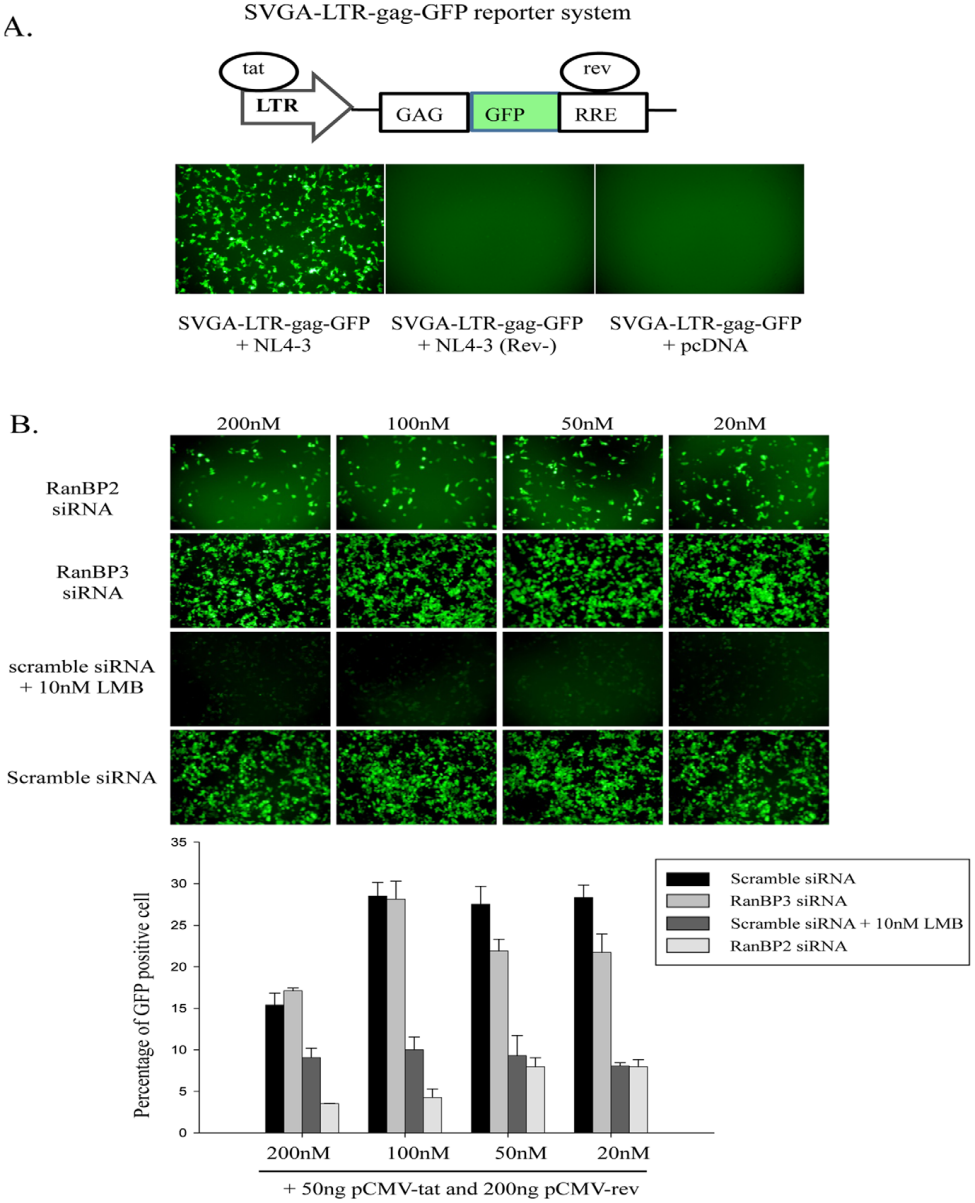
RanBP2 depletion impairs biological activities of ectopically expressed Tat and Rev HIV proteins. (**A**) Schematic diagram of Tat/Rev responsive SVGA-LTR-gag-GFP cells is shown. The cells were transfected with either 50 ng NL4-3 plasmid, or 50 ng NL4-3 (Rev-). Fluorescent images were captured 48 h post transfection. (**B**) SVGA-LTR-gag-GFP reporter cells were transfected either with RanBP2 siRNA, RanBP3 siRNA (negative control) or scrambled siRNA (control). 48 h later, the cells were then co-transfected with 50 ng pCMV-Tat and 200 ng pCMV-Rev. In parallel, Leptomycin B was used as positive control and was added 4 h after the transfection of reporter cells (pre-transfected with scrambled siRNA) with pCMV-Tat and pCMV-Rev. The images were captured and GFP positive cells were analyzed by flow cytometry 48 h post plasmid transfection.

Rev protein is an essential viral protein that regulates the temporal HIV-1 gene expression. Earlier studies have shown that RanBP2 regulates the nucleo-cytoplasmic trafficking of HIV-1 Rev protein [13]–[14]. Although impressive, these experimental observations, however, were based on the usage of plasmid expression vectors via transfection and were not substantiated by HIV infection studies. To address whether the function of HIV-1 Rev protein is impaired in RanBP2 depleted cells, we have established SVGA-LTR-gag-GFP reporter cells wherein the expression of gag-GFP fusion product under HIV-LTR promoter is regulated by both Tat and Rev proteins. The stable monoclonal LTR-gag-GFP-RRE reporter cells were transfected with either the wild type HIV provirus NL4-3, or the HIV provirus with mutant Rev, pMRev(-) as a control, and monitored for GFP expression. The GFP expression was observed only in cells with wild type HIV provirus (**Fig. 2A**). We then used these reporter cells to test the role of RanBP2 in Rev-mediated mRNA export. SVGA-LTR-gag-GFP reporter cells transfected with either RanBP2 siRNA, RanBP3 siRNA or scrambled siRNA were co-transfected 48 hrs later with pCMV-Tat and pCMV-Rev expression vectors, and monitored for the GFP expression. We observed a profound decrease in GFP expression in RanBP2 siRNA transfected reporter cells compared to RanBP3 (negative control) or scrambled siRNA ones (**Fig. 2B**). The CRM1, a host nuclear shuttling protein, is involved in Rev-mediated nuclear export of HIV mRNAs. Leptomycin B (LMB), a known inhibitor of CRM1-mediated mRNA export pathway [54], was used as a positive control and revealed a robust inhibitory effect on the GFP expression (**Fig. 2B**).

**Figure 3:**
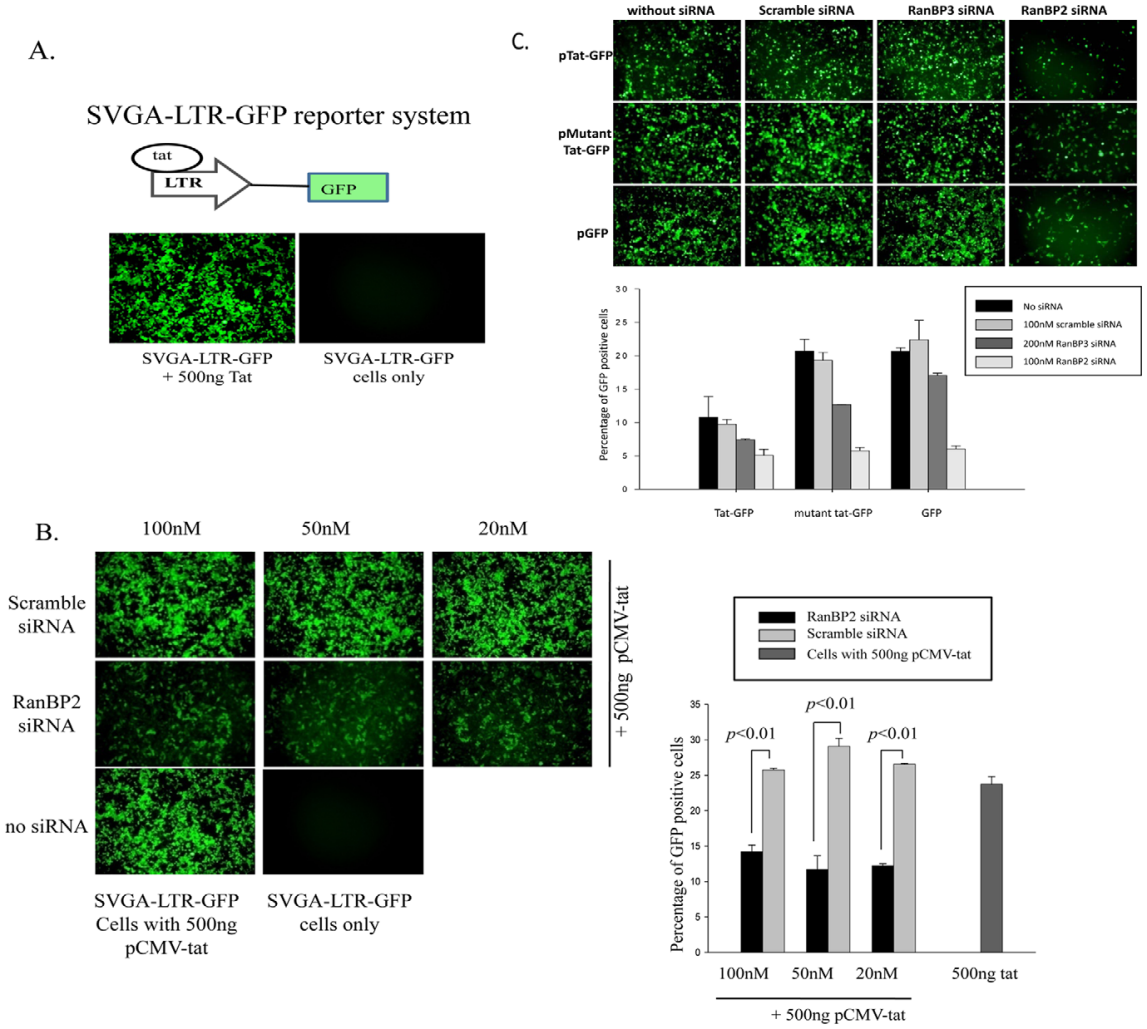
RanBP2 depletion inhibits global ectopic gene expression. (**A**) Schematic diagram of Tat responsive SVGA-LTR-GFP reporter cells is shown. Cells were either transfected with 500 ng pCMV-Tat or pcDNA vector (control) and images were captured 48 h after transfection. (**B**) SVGA-LTR-GFP reporter cells were transfected either with series of concentrations of RanBP2 siRNA or scrambled siRNA. 48 h later, the cells were transfected with 500 ng pCMV-Tat. After 48 h the images were captured and GFP positive cells were analyzed by flow cytometry. (**C**) SVGA cells were transfected either with 100 nM RanBP2 siRNA, 200 nM RanBP3 siRNA or 100 nM scrambled siRNA. 48 h later, the cells were then transfected either with 100 ng pEGFP, 100 ng pTat-GFP or 100 ng pΔTat-GFP plasmids respectively. The GFP expression was examined 48 h later by capturing the images and analysis by flow cytometry.

Since GFP expression in SVGA-LTR-gag-GFP reporter cells is dependent on both Tat-mediated LTR activation and Rev-mediated mRNA export, the above results indicate that RanBP2 knockdown must have blocked either Tat-mediated transactivation of LTR promoter or Rev-mediated mRNA export. To further investigate whether RanBP2 depletion attenuates Tat-mediated transactivation of LTR-promoter, we used SVGA-LTR-GFP reporter cells wherein the GFP expression is dependent on Tat protein, but independent of Rev, and observed robust Tat-dependent GFP expression (**Fig. 3A**). After validating the reporter system, we transfected reporter cells either with RanBP2 siRNA or scrambled siRNA, followed by transfection with pCMV-Tat expression vector 48 hr later. Intriguingly, we found that Tat-driven GFP expression was profoundly inhibited in RanBP2-depleted but not in scrambled siRNA transfected reporter cells (**Fig. 3B**).

Inhibition of Tat-dependent LTR-GFP expression in RanBP2-depleted cells led us to investigate the mechanism further. To exclude the possibility that functional Tat protein do get expressed in the cytoplasm of RanBP2-depleted cells but is inhibited at the nuclear import level, we used Tat-GFP expression vector wherein Tat is expressed as a fusion protein with GFP to test the hypothesis. SVGA cells transfected with either RanBP2 siRNA, RanBP3 siRNA or scrambled siRNA, were transfected 48 hrs later with Tat-GFP expression vector and monitored for GFP expression. GFP expression was dramatically decreased in RanBP2 ablated but not in RanBP3 or scrambled siRNA transfected cells (**Fig. 3C**). This observation demonstrated that RanBP2 ablation inhibited Tat expression. We then sought to investigate whether the inhibitory effect was Tat-specific or universal. We used pΔTat-GFP (GFP gene fused with mutant Tat gene wherein 48-56 amino acids were deleted) and pEGFP expression vector systems to validate the above findings. Unambiguously, we observed pronounced GFP expression decline in RanBP2-ablated SVGA cells compared to control siRNAs (**Fig. 3C**). These observations inferred that, rather than specific inhibition of Tat expression, RanBP2 knockdown inhibits global ectopic gene expression. Taken together, by using SVGA-LTR-gag-GFP-RRE and SVGA-LTR-GFP reporter systems, we serendipitously found that RanBP2 was universally indispensible in ectopic gene expression.

**Figure 4:**
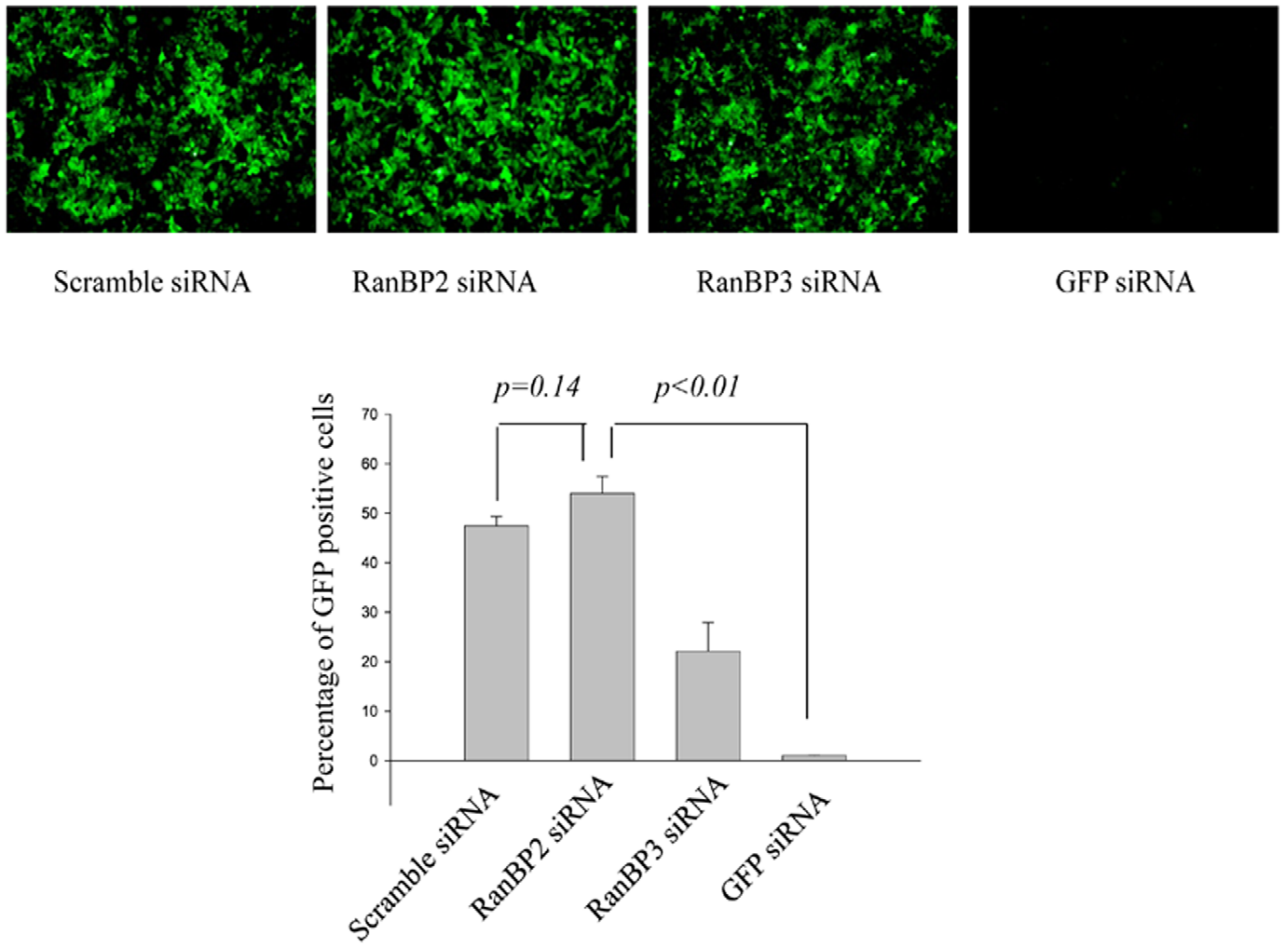
RanBP2 depletion does not inhibit GFP expression in SVGA-GFP transgenic cells. Stable SVGA-GFP cells were transfected overnight either with 100 nM RanBP2 siRNA, 200 nM RanBP3 siRNA (negative control), 100 nM scrambled siRNA (control), or 100 nM GFP siRNA as a positive control. The cells were then followed up for 5 days. On day 3 images were captured and the GFP positive cells were analyzed by flow cytometry.

We further sought to investigate the specific role RanBP2 plays in the ectopic gene expression and whether it regulates stable GFP expression and is involved in the nuclear export of the mRNAs encoded from plasmid vectors. To test the hypothesis and rule out the transfection based reporter expression (transient expression) influence, SVGA-GFP transgenic cells with stable GFP is expression, were chosen to examine whether RanBP2 knockdown interfered with the nuclear export of GFP mRNA (absence of GFP expression). SVGA-GFP cells were transfected either with RanBP2 siRNA, RanBP3 siRNA (negative control), scrambled siRNA or GFP siRNA as a positive control. The siRNAs transfected cells were cultured for 5 days and examined for GFP fluorescence every day. Surprisingly, the GFP expression in RanBP2-depleted cells was similar to RanBP3 or scrambled siRNA transfected cells, while GFP siRNA dramatically inhibited GFP expression since the second day of siRNA transfection. The images were captured and the GFP-positive cells were quantified by flow cytometry on 3^rd^ day after the siRNA transfection (**Fig. 4**). These results clearly demonstrated that RanBP2 siRNA did not interfere with the nuclear export of mRNA but might be playing a role in the nuclear import of DNA.

### RanBP2 ablation does not impair nuclear export of HIV mRNAs or expression of HIV early gene products

RanBP2 ablation had no impact on the GFP mRNA expression and its translation in the stable cells (**Fig. 4**). However, it remains yet to be elucidated whether RanBP2 is essential in Rev-mediated nuclear export of unspliced or partially spliced HIV mRNA species. To corroborate whether RanBP2 regulates Rev-mediated nuclear export of HIV-1 mRNA, we took advantage of the latently HIV-1 infected cell system – THP89 cells. THP89 is a monocytic cell line latently infected with recombinant p89.6 HIV-1 reporter virus wherein the GFP gene is inserted in the viral genome [48]. These latently HIV-1 infected cells are transcriptionally silent and show no viral mRNA transcripts and their translated products unless reactivated by either TNF-α, histone deacetylase inhibitor Trichostatin A (TSA, or protein kinase C (PKC) activator bryostatin [48], [51], [52]. To perform reactivation studies on latent THP89 cells, we used bryostatin, a drug that is recently shown by us to be very potent in reactivation of latent HIV-1 [52]. Upon bryostatin mediated reactivation, the viral mRNAs are exported from the nucleus into the cytoplasm either for translation into proteins including the GFP or as genomic copies for packaging into viral particles.

**Figure 5:**
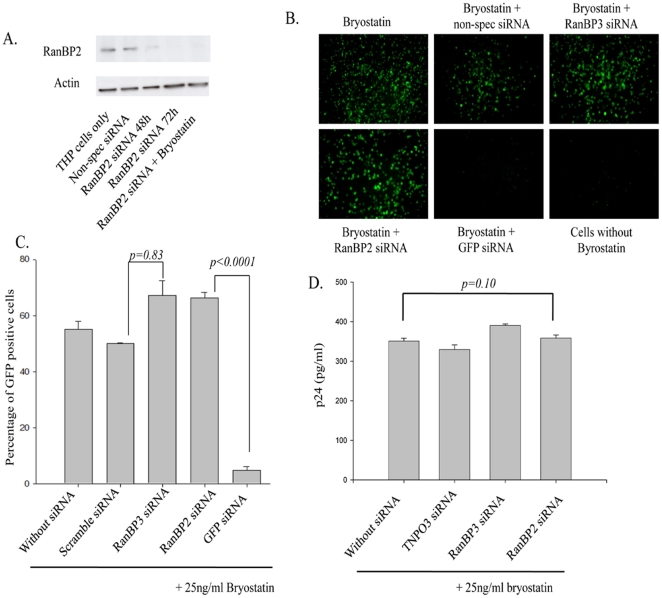
RanBP2 depletion does not interfere with nuclear export of viral mRNA species. (**A**) Latently HIV infected monocytic (THP89) cells were transfected with 100 nM RanBP2 siRNA and total proteins were extracted 48 or 72 h post transfection. Western blot analysis revealed the levels of RanBP2 protein. RanBP2 level in cells that were transfected with 100 nM RanBP2 siRNA followed by bryostatin treatment were also examined the performance on viral activity. THP89 cells either transfected with scramble siRNA or left untransfected were used controls. (**B–D**) THP89 cells were first transfected either with RanBP2 siRNA, RanBP3 siRNA, scrambled siRNA or GFP siRNA as a positive control. 48 h later, the cells were reactivated with 25 ng/ml bryostatin (potent reactivator of latent HIV infection). The GFP expression was examined 48 hrs after bryostatin treatment. (**B**) The Images were captured and (**C**) GFP positive cells were analyzed by flow cytometry. (**D**) Supernatants collected in (**B**) were analyzed for p24 levels by ELISA.

THP89 cell line is thus an ideal system to study whether RanBP2 is directly involved in the nuclear export of viral mRNAs and is superior to transfection and infection experiments wherein viral DNA nuclear import and integration are additional steps to be suspected before concluding Rev function. Before performing the actual experiments, we first checked the efficacy of RanBP2 siRNA in depleting the protein in THP89 cells. As expected, RanBP2 protein was profoundly depleted by the specific siRNA and was not detectable 72 hr post siRNA transfection and such effect was not seen in control siRNAs (**Fig. 5A**). In order to study the effect of RanBP2 on mRNA expression, we performed siRNA inhibition studies in the presence or absence of bryostatin and observed that bryostatin had no interference with RanBP2 siRNA performance (**Fig. 5A**). Thereafter, THP89 cells were first transfected either with RanBP2, RanBP3 siRNA or scrambled siRNA. In parallel, GFP siRNA was used as a positive control. 48 hrs after siRNA transfection, the cells were treated with bryostatin.

Viral reactivation in bryostatin treated THP89 cells was monitored by GFP expression (fluorescence microscopy and flow cytometry), and viral p24 antigen in the culture supernants by ELISA. Fluorescence microscopy and flow cytometry analysis revealed no difference among RanBP2- and RanBP3-depleted or scrambled siRNA transfected cells but profound decline in fluorescence was seen in GFP siRNA transfected cells (positive control). Flow cytometry data was validated by p24 antigen levels (a marker for productive HIV infection) wherein no effect of RanBP2 depletion was seen (**Fig. 5B–D**). Overall, these results unambiguously demonstrate that RanBP2 does not play a role in the nuclear export of HIV-1 mRNA species via Rev and this is in contrast to the earlier studies [13], [14].

### RanBP2 depletion inhibits HIV-1 replication

**Figure 6:**
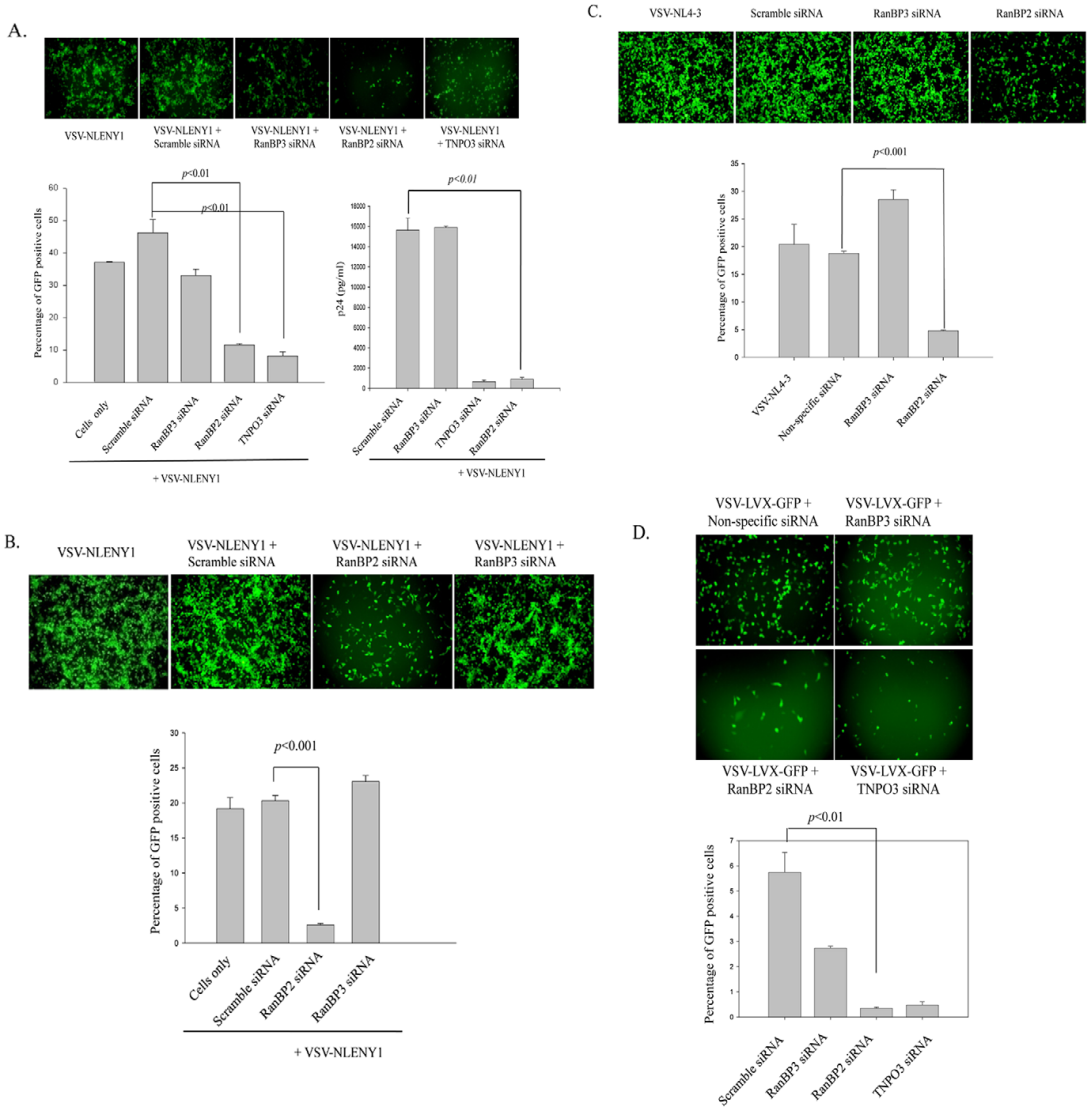
RanBP2 depletion inhibits HIV-1 replication and lentiviral vector gene expression. (**A**) Magi cells were transfected either with RanBP2 siRNA, RanBP3 siRNA (negative control), TNPO3 siRNA (positive control) or scrambled siRNA (control). 48 h later, the cells were transduced with 200 ng/ml p24 equivalent VSV-NLENY1 (recombinant HIV virus containing YFP) for 16 h. The fluorescent images were taken 48 h post transduction followed by collection of the culture supernatants for p24 (ELISA). The YFP positive cells were analyzed by flow cytometry (p<0.01). (**B**) SVGA cells were transfected either with RanBP2 siRNA, RanBP3 siRNA or scrambled siRNA. 48 h later, the cells were transduced with 200 ng/ml p24 equivalent VSV-NLENY1 for 16 h. The fluorescent images were captured 48 h post transduction by digital camera (Nikon) and YFP positive cells were analyzed by flow cytometry (p<0.001). (**C**) SVGA-LTR-GFP reporter cells were transduced either with RanBP2 siRNA, RanBP3 siRNA (negative control), scramble siRNA or TNPO3 siRNA as a positive control. 48 hrs later, the cells were then transduced with 200 ng/ml p24 equivalent VSV-NL4-3. Images were captured 48 h after transduction and the GFP positive cells were analyzed by flow cytometry (p<0.01). (**D**) SVGA cells were transfected either with RanBP2 siRNA, RanBP3 siRNA, TNPO3 siRNA or scrambled siRNA. 48 h later, the cells were transduced with VSV-G pseudotyped lentiviral vector. The fluorescent images were captured 48 h after transduction and GFP positive cells were analyzed by flow cytometry (p<0.01).

In order to investigate the role of RanBP2 in HIV-1 replication, we performed RNAi studies on Magi cells, a Hela cell line that expresses CD4, CCR5, and CXCR4 receptors, followed by the viral infection. As a positive control, we used siRNA targeting transportin 3 (TNPO3), a known host factor that is essential in HIV-1 replication and promotes viral DNA nuclear import [44], [45], [55]. Magi cells transfected either with RanBP2 siRNA, RanBP3 siRNA (negative control), scrambled siRNA (control), or TNPO3 siRNA (positive control), and were further transduced with VSV-G pseudotyped NLENY1, a recombinant HIV-1 expressing YFP [51]. The rationale of using VSV-HIV particles, as against the wild type virus, was to achieve homogenous and rapid infection within two days in order to obtain consistent end points in the dividing cells (different cell types). The YFP-gene in NLENY1 is cloned between the HIV env- and -nef genes using IRES (internal ribosome entry site sequence) and hence, will encode partially spliced Env-GFP mRNA (translates into two separate proteins) and expression will be dependent on functional Rev-protein. The end point after infection was monitored by YFP expression in the infected cells as its expression was dependent on the functional Rev-protein. RanBP2 siRNA similar to TNPO3 siRNA robustly diminished the YFP expression as well as viral p24 antigen levels compared to the RanBP3 or scrambled siRNA controls (**Fig. 6A**).

To corroborate that the inhibitory effect conferred by RanBP2 siRNA was not cell type specific, we further validated our findings by using a different cell system – SVGA cells, the astrocytic cell line commonly used in our LTR-reporter assays. Remarkably, RanBP2 depletion in SVGA cells similar to Magi cells revealed a significant decrease in the YFP-positive cells compared to RanBP3 or scrambled siRNA transfected cells (**Fig. 6B**). Further, we took advantage of Rev-independent expression of the HIV early proteins to investigate whether RanBP2-depleted cells could express functional Tat protein. We used SVGA-LTR-GFP reporter cells in combination with VSV-pseudotyped NL4-3 virus. The hypothesis is that VSV-HIV infection in RanBP2 depleted cells synthesizes viral mRNAs, and the transportation and expression of Tat mRNA should be independent of Rev function but dependent on the cellular mRNA transport machinery. Should the Tat expression in the above be undetectable, the possible reason would be that VSV-HIV infection in RanBP2-depleted cells has an impeded viral DNA nuclear import and hence no genomic viral integration, transcription and early viral proteins synthesis. SVGA-LTR-GFP reporter cells were depleted for RanBP2, 48 hrs later followed by transduction with VSV-HIV and examined for Tat-driven LTR mediated GFP expression. VSV-HIV transduced RanBP2-depleted SVGA-LTR-GFP cells showed profound decline in GFP positive cells compared to wild type cells or cells transfected with either RanBP3 siRNA or scrambled siRNA (**Fig. 6C**). Since Tat expression is a downstream event of nuclear entry of viral DNA, its low expression, obviously, can be explained by the impeded nuclear import of the viral DNA in RanBP2 depleted cells.

In order to confirm the role of RanBP2 in viral regulation, we further validated our data using a HIV-1 derived lentiviral vector wherein reporter gene (GFP) expression is no more controlled by HIV proteins. We used a pLVX-GFP expressing lentiviral vector that does not require any HIV proteins to express GFP and was packaged with VSV-G envelope. SVGA cells transfected 48 hrs earlier either with RanBP2 siRNA, RanBP3 siRNA, scrambled siRNA or TNPO3 siRNA, were transduced with VSV-G pseudotyped lentiviral vector. When monitored by fluorescence microscopy and flow cytometry, it was observed that RanBP2 siRNA and TNPO3 siRNA, similar to the earlier experiments using VSV-HIV (**Fig. 6A, B**), dramatically inhibited the lentiviral vector mediated GFP expression compared to RanBP3 siRNA or scrambled siRNA (**Fig. 6D**). These results provide evidence that RanBP2, a host nuclear membrane component, is critical in establishing productive HIV infection. The above findings also revealed that RanBP2-depletion impairs HIV life cycle upstream of the early viral protein expression but at least not at the viral entry level as VSV-pseudotyped virus particles entry is receptor independent.

### RanBP2 depletion inhibits nuclear import of HIV-1 DNA (PIC)

**Figure 7:**
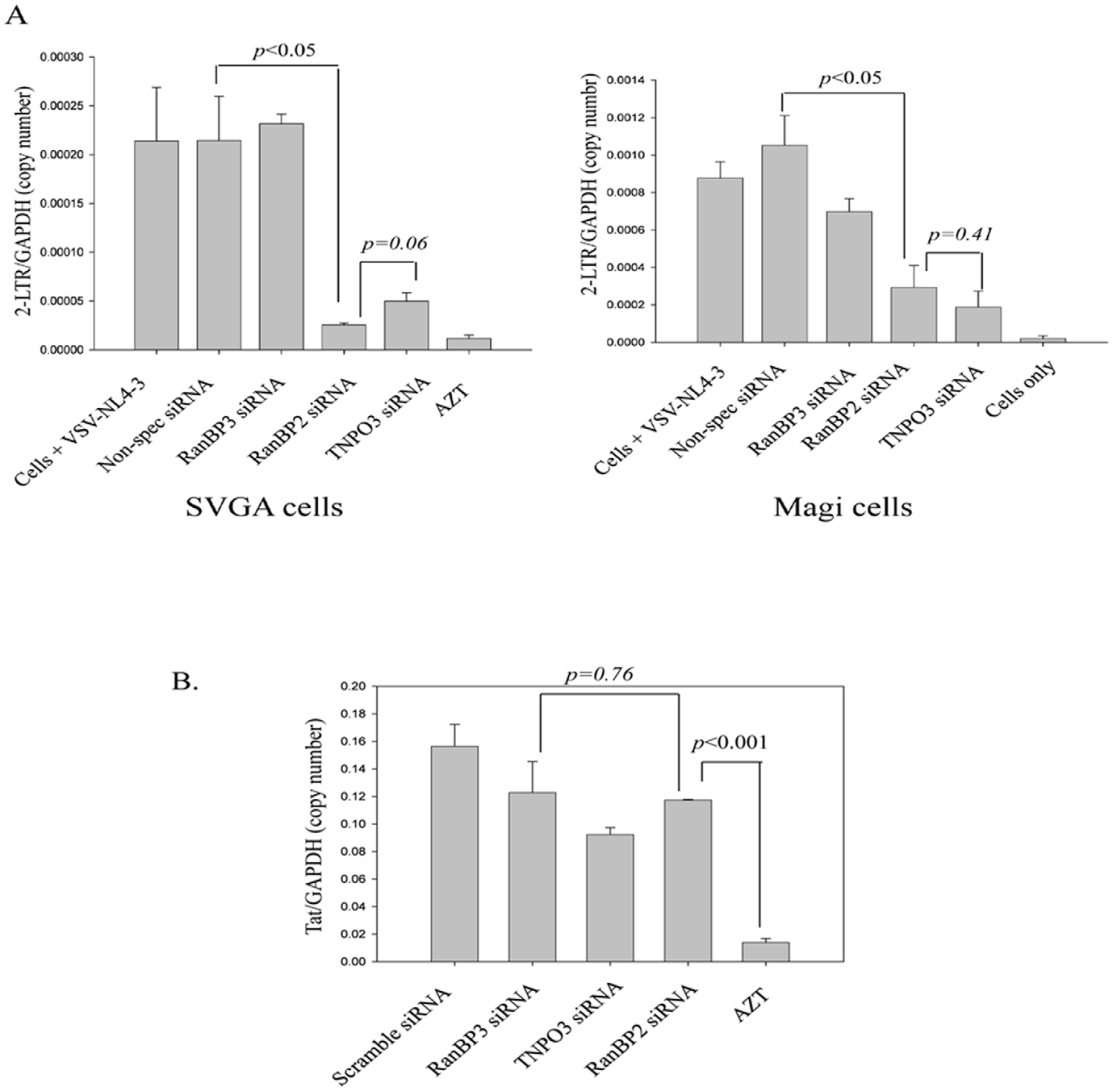
RanBP2 depletion inhibits 2-LTR circle formation (HIV DNA) but not viral reverse transcription. SVGA and Magi cells were transfected either with RanBP2 siRNA, RanBP3 siRNA (negative control), scrambled siRNA or TNPO3 siRNA as a positive control. 48 h later, the cells were transduced with 200 ng/ml p24 equivalent VSV-NL4-3 (HIV). AZT, a reverse transcriptase inhibitor, was used as a positive control to inhibit the reverse transcription and added 30 min before VSV-HIV transduction. (**A**) Total DNA was extracted 24 h after VSV-HIV transduction and 2-LTR levels were examined by real-time PCR. (**B**) Total DNA from SVGA cells was extracted 12 hrs after VSV-HIV transduction and the viral reverse transcription was examined by real-time PCR.

NPC plays a critical role in nuclear import of HIV DNA and in a global siRNA screening study RanBP2 has been shown to be involved in the viral DNA import [40]. Also, RanBP2 is involved in cellular nucleo-cytoplasmic trafficking as shown in the earlier studies [13], [14], [40]. After validation of non-involvement of RanBP2 in the viral mRNAs export we then sought to investigate whether it promotes nuclear entry of the viral DNA. To study HIV-DNA nuclear import in absence of RanBP2, we took advantage of 2-LTR circles of viral DNA, a marker for the nuclear import of HIV-1 DNA [56]–[58]. SVGA cells were first transfected either with RanBP2 siRNA, RanBP3 siRNA, scrambled siRNA or TNPO3 siRNA as a positive control. The siRNA transfected cells, 48 hrs later were transduced with VSV-G pseudotyped HIV, and the total DNA was extracted 24 hrs later, (viral DNA enters the nucleus around 16 hrs post infection [56]). The levels of 2-LTR circles in the extracted DNA were measured by using real time PCR. There was a profound decrease in the levels of 2-LTR circles in RanBP2- and TNPO3 (positive control)-depleted cells but not in RanBP3 (negative control)-depleted or scrambled siRNA transfected cells (**Fig. 7A**).

To corroborate the above results we utilized another cell system Magi cells and performed similar knockdown studies. Strikingly, RanBP2-depleted Magi cells revealed reduced levels of the 2-LTR circles similar to TNPO3-depleted cells (positive control) but not RanBP3 or scrambled siRNA controls (**Fig. 7A**). To rule out any inhibitory effect of RanBP2 siRNA on viral reverse transcription, we further examined the levels of viral late reverse transcription products 12 hrs after the virus infection that would reflect the total viral DNA synthesized in RanBP2 depleted cells and the controls. Indeed, we observed that the treatment with RanBP2 siRNA or control siRNA does not show any inhibitory effect on the total reverse transcribed viral DNA, whereas AZT treated cells (positive control that inhibits the viral reverse transcriptase) revealed absence of RT-product (**Fig. 7B**). The above results suggest that RanBP2 knockdown does not interfere at the viral entry and the reverse transcription steps.

All together, in two different systems, reduction in 2-LTR DNA circles formation but occurrence of equal amounts of reverse transcription products in RanBP2-depleted cells demonstrated that RanBP2 plays a pivotal role in nuclear import of HIV DNA (PIC). Taken together, our multiple lines of evidence confirm that RanBP2 is a required host factor for HIV-1 replication and promotes the nuclear entry of HIV-DNA but not the nuclear export of viral mRNA species.

## Discussion

In this study, we investigated the role of RanBP2 and found that it is an essential host factor for HIV-1 infection and is involved in promoting the nuclear import of HIV-1 DNA. The nuclear export of viral mRNAs was unaffected in RanBP2 depleted cells. Further, we showed that RanBP2 is indispensable in ectopic gene expression via plasmid vectors and transduction of HIV/lentiviral particles, substantiating its role in the nuclear DNA import.

To investigate the specific steps blocked by RanBP2 depletion, sequentially, we first examined whether the function of HIV-1 Rev protein is impaired. THP89 cells, that are latently infected with HIV-1 and can be reactivated upon treatment with either bryostatin or TNF-α, provided an excellent model to study the nuclear export of HIV-1 mRNAs. Upon bryostatin treatment, HIV-1 transcription is activated and subsequently the synthesized viral mRNAs are exported from the nucleus into the cytoplasm to allow the viral protein synthesis and completion of the viral life cycle. Thus, impaired viral replication in RanBP2-depleted THP89 cells upon treatment with bryostatin can imply a deficient nuclear export of the viral mRNA species and absence of the translated proteins (GFP/p24). Our data clearly show that RanBP2 depletion does not affect HIV-1 replication in reactivated THP-89 cells (**Fig. 5**), suggesting that RanBP2 does not function in the nuclear export of viral mRNAs.

Since HIV-1 Rev shuttles between the cytoplasm and the nucleus and is responsible for the nuclear export of unspliced and partially spliced viral mRNAs, our data clearly demonstrate that RanBP2 does not interfere in the nuclear-cytoplasmic shuttling of HIV Rev protein, as no viral replication block was found in our latent HIV–reactivation model. This is contrary to earlier reports demonstrating that nuclear import of HIV-1 Rev is mediated mainly by the transportin, a nuclear import receptor, and that depletion of RanBP2 inhibits all transportin - mediated nuclear import and causes accumulation of Rev in the cytoplasm [13]. Similarly, another study shows that depletion of RanBP2 mislocalizes CRM1, the host export receptor that mediates HIV-1 Rev nuclear export and subsequently causes accumulation of Rev in the nucleus [14]. In both the studies, Rev-BFP plasmid was transiently transfected into RanBP2 depleted cells and percentages of nuclear localized- or cytoplasmic localized-Rev positive cells were measured. Therefore, we argue that the inherent weaknesses in both the studies lie in the experimental models itself and the conclusion extended may not be appropriate in transient ectopic gene expression system. In contrast, global impact of RanBP2 depletion in ectopic gene expression (**Fig. 2** **,** **3**) but not in stable GFP expression system is clearly shown in our study (**Fig. 4**). We have provided exquisite three-pronged evidences of RanBP2-independent Rev-function using acute HIV and lentiviral vector infection (**Fig. 6**), latent infection model (**Fig. 5**) and Rev-dependent and -independent reporter systems (**Fig. 6C**).

One caveat in our observations is that RanBP2 promotes nuclear import of HIV-1 DNA. The levels of 2-LTR circles, a marker for the nuclear import of viral genomic DNA, were investigated and revealed that RanBP2 depletion markedly decreased the levels of 2-LTR circles but not the viral reverse transcription product in infected cells (**Fig. 7**), suggesting that RanBP2 depletion impedes the nuclear import of viral DNA (PIC). We further verified the post-viral DNA integration consequences in RanBP2-depleted SVGA-LTR GFP cells infected with VSV-HIV. Similarly, RanBP2 depletion impaired post integration consequences such as Tat-dependent but Rev-independent GFP expression (**Fig. 6C**), suggesting that the blockade occurred before the viral DNA integration and transcription steps.

Studying the mechanism of nuclear entry of HIV-1 DNA has attracted great attention. In earlier studies, several HIV-1 viral components that promote nuclear import of HIV-1 PIC were identified such as HIV-1 IN, Vpr, MA and cPPT-CTS, (central DNA flap) and all have been found to carry karyophilic signals [59]–[64]. Besides, host factors such as importin α, importin β, importin 7, and transportin – SR2 (TNPO3) have been described to be responsible for the nuclear import of HIV-1 DNA [44], [65]–[68]. In our study, we did not explore further how RanBP2 interacts with HIV-1 PIC-importin cargo complex, but it could be interesting to pursue in the future. It would be intriguing to identify the viral components, host importins and domains of RanBP2 that are responsible for the interactions. Designing small RanBP2 mimetic peptides that competitively interact with HIV-1 PIC and thus disrupt the interaction between the cargo complex and RanBP2 would be an alternative strategy to inhibit HIV-1 replication.

**Figure 8:**
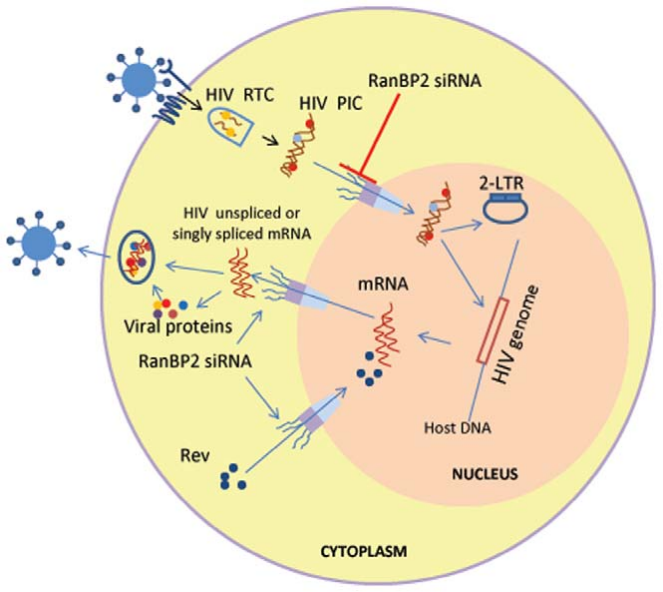
Schematic diagram illustrating role of RanBP2 in HIV-1 DNA (PIC) nuclear import. HIV-1 entry follows partial uncoating of HIV particles with concomitant viral RNA reverse transcription into cDNA and assembly into PIC with viral proteins MA, Vpr and integrase. The PIC import into the nucleus is facilitated by nuclear membrane component RanBP2. RanBP2 depletion via RNAi inhibits PIC import into the nucleus. The PIC import is detected by the 2-LTR circles (marker of HIV DNA nuclear import). RanBP2 is not involved in HIV-Rev protein nuclear import/export as demonstrated by HIV replication (GFP expression as a marker or viral p24 antigen) seen in HIV latent THP89 model (HIV reactivation by bryostatin).

In conclusion, our study provides evidence that RanBP2 is a required host factor for HIV-1 replication and promotes the nuclear import of viral DNA but not the nuclear export of the viral mRNA species or nucleo-cytoplasmic trafficking of Rev (**Fig. 8**).

## Materials and Methods

### Cell culture, transgenic cells, and reagent

Transgenic cell lines were established as previously described [46]. We established SVGA-GFP, SVGA-LTR-GFP, SVGA-LTR-gag-GFP cells for the present study [46], [47]. SVGA, a human fetal astrocytic cell line, SVGA-LTR GFP or -LTR-gag-GFP RRE reporter cells, 293 HEK cells, and Magi (CD4/CCR5) cells [47] were all maintained in DMEM (Gibco BRL life technologies, NY, USA) containing 10% Fetal Bovine Serum (FBS), 100 U/ml penicillin and 100 µg/ml streptomycin. Latently HIV infected monocytic cell line THP89 [48] was maintained in RPMI supplemented with 10% FBS and antibiotics. Leptomycin B and AZT were purchased from Sigma.

### Plasmids and Viral Constructs

HIV-1 proviral DNA NL4-3 from Dr. Malcolm Martin [49], pMrev(-) from Dr. Reza Sadaie [50], and VSV-G expression plasmids were obtained through NIH AIDS Research and Reference Reagent Program (NIAID, Bethesda, USA). The recombinant reporter HIV-1, NLENY1 was created by inserting the YFP gene between envelope and Nef in NL4-3 proviral DNA [51]. HIV-1 derived lentiviral vector pLVX was purchased from Clontech. The full length HIV-Tat and -Rev from HXB-2b were cloned in pcDNA vector, respectively and sequences were verified by DNA sequencing [46]. Further, Tat and a deletion mutant of Tat (ΔTat) in which amino acids 48-56 were deleted by PCR, were inserted in frame upstream of GFP gene in the pEGFP expression vector (Clontech) [46]. The HIV long terminal repeat (LTR) driven GFP construct was made by replacing CMV promoter in the pEGFP vector with LTR promoter at SalI and SmaI [46]. pQBI-LTR-gag-GFP was purchased from Quantum Biotechnologies Inc.

### HIV/Lentiviral vector pseudotyping and infection

VSV-G pseudotyped viral particles were prepared by using HEK 293 cells as described earlier [48]. Briefly, HEK 293 cells were seeded in 100 mm cell culture dishes. 24 hrs later cells were transfected with either 17 µg pNLENY1 or pNL4-3 together with 4 µg pVSV-G using Lipofectamine 2000. In order to pseudotype lentiviral vector, cells were transfected with 10.0 µg pLVX, 3.0 µg pCMV-Tat, 8.0 µg pGag-Pol, 4.0 µg pCMV-Rev, 2.5 µg pVpr and 4.0 µg pVSV-G using Lipofectamine 2000. Transfection of the plasmid expression vectors were performed as described previously [47]. The next day, transfected cells were cultured in fresh DMEM medium containing 10% FBS. 72 hours post transfection; the supernatants were harvested and centrifuged at 300× g for 15 min. The pseudotyped viral stocks were titrated for HIV p24 antigen (ZeptoMetrix, NY, USA) [52] and one ml aliquots were stored at −80°C. Virus inoculums at 200 ng p24 concentrations were then used in transduction experiments. Magi (CD4/CCR5) cells, SVGA cells, and SVGA-LTR-GFP reporter cells were transduced with VSV-G pseudotyped NLENY1, or NL4-3, or HIV-1 derived lentiviral vector. Cells were seeded in 12-well culture plates, and were transduced next day with VSV pseudotyped NLENY1 or NL4-3 or lentiviral vector for 16 hours. The infected cells were then washed twice and cultured in fresh medium for another 24 hours. The GFP expression was monitored and the images were captured by a digital camera in a fluorescent microscope (Nikon). GFP positive cells were measured by flow cytometry (Beckman Coulter) and the viral activity was measured by p24 ELISA on the harvested culture supernatants [48], [52].

### RNA interference

RanBP2 siRNA (sequence 5′- CCGUUUUGGUGAGUCAACAtt - 3′, siRNA ID s11773, cat no. 4390824), positive control GFP siRNA (cat no. AM4626) and negative control RanBP3 siRNA (sequence 5′- GGAAGCCUGUGAGAAAAAAtt - 3′, siRNA ID 19354, cat no. AM16708) were purchased from Ambion. TNPO3 siRNA (positive control) was designed by web-based siRNA converter programs (Ambion, Austin, TX) and sequences were synthesized by Dharmacon. TNPO3, sense 5′-CGACAUUGCAGCUCGUGUAUU-3′, and antisense 5′-UACACGAGCUGCAAUGUCGUU-3′; Scrambled siRNA from Dharmacon was used as a control. For siRNA transfection, Lipofectamine 2000 (Invitrogen) was used according to the manufacturer's guidelines.

### siRNA transfection and reactivation of latent HIV

THP89 cells, a latently HIV-1 infected monocytic cell line [47], [48], were seeded into 12-well culture plates. 48 hrs later, cells were transfected with RanBP2 siRNA, RanBP3 siRNA (negative control), TNPO3 siRNA (positive control), scramble siRNA (control), or GFP siRNA (positive control). The basal viral activity in THP89 cells was barely detectable with complete absence of GFP-expression and p24 [48], [52]. Viral reactivation was performed 48 hours post siRNA transfection with 25 ng/ml bryostatin (Enzo life sciences). Images were captured followed by collection of the culture supernatants for p24 analysis and GFP positive cells were measured by Flow Cytometry after 48 hrs of bryostatin treatment [52].

### Flow cytometry

The percentages of GFP positive cells were measured in a Epics XL FACS flowcytometer with Expo32 software (Beckman Coulter, Inc., FL, USA). The cells were trypsinized, followed by washing with PBS and finally resuspended in 1 ml PBS containing 1% FBS. In a situation of reactivated THP89 cells or cells that were transduced with VSV-NLENY1 or VSV-NL4-3 viruses, 3% paraformaldehyde was used to fix the cells for 30 min at 4°C followed by washing with PBS.

### Western Blotting

Magi, SVGA, or THP89 cells were transfected either with RanBP2- or RanBP3-siRNA and were harvested 72 hrs post transfection. The cells were lysed in RIPA buffer (Sigma) and total proteins in equal amounts were subjected to SDS-PAGE. Proteins were then transferred to polyvinylidene difluoride (PVDF) membranes (pore size 0.2 µm, BioRad). For RanBP2 detection, 5% SDS-PAGE gel was used to separate the total cellular proteins and followed by transfer to PVDF membrane at 30 V for 24 hours, using transfer buffer containing 10% methanol with 0.5% SDS. The membranes were blocked for 1 hr at room temperature with blocking buffer (5% nonfat dry milk, 0.1% Tween 20 in PBS). The blotted membranes were probed overnight at 4°C with rabbit polyclonal antibody against RanBP2 (1∶500 dilutions, abcam) in blocking buffer. After three washes with PBS/0.1% Tween 20, the membranes were incubated with anti-rabbit IgG conjugated to horseradish peroxidase secondary antibody (1∶10’000 dilution, sigma) in blocking buffer for 1 hr at room temperature. The membranes were washed three times with PBS/0.1% Tween 20 and developed using chemiluminescence (ECL) detection kit (GE Healthcare). As an internal control, actin was probed with mouse monoclonal antibody against actin (1∶5000 dilution, Sigma) and then with anti-mouse IgG (1∶10’000 dilution, Sigma). To detect the level of RanBP3, we used rabbit polyclonal antibody against RanBP3 (1∶1000 dilution, abcam). The protein bands were normalized to β-actin (1∶10000) and were quantified using ImageJ software (v 1.41; NIH) [52].

### DNA extraction and Real time PCR

In each case, the cells were trypsinized and washed twice with PBS and centrifuged at 300×g for 15 min. One ml DNAzol (Invitrogen) was added to cell pellet and 0.5 ml 100% ethanol (Sigma) was used to precipitate DNA. The precipitated DNA was then washed with 75% ethanol twice and dissolved in 8 mM NaOH (pH 8.0) [47]. PCR amplification was performed in a 25 µl reaction mix containing 25 pmol of each primer, 2 X SYBR mix (BioRad) and 1 µl DNA template. The cycle program used was 95°C/10 min followed by 95°C/10 s, 60°C/30 s, and 72°C/30 s for 39 cycles. Reactions were run on CFX96 real time PCR system (Bio-RAD) and data was collected and analyzed using Bio-RAD CFX Manager Software v 1.1. Ct values were calculated for each gene and normalized relative to GAPDH expression. Fold expression from untreated control or ΔΔCt was calculated using Paffafl method.

The following primers were used:

2-LTR forward: 5′-CCCTCAGACCCTTTTAGTCAGTG-3′,

2-LTR reverse: 5′-TGGTGTGTAGTTCTGCCAATCA-3′;

GAPDH forward: 5′- GTCAAGGCTGAGAACGGGAAGC-3′,

GAPDH reverse: 5′- AGGGATCTCGCTCCTGGAAGATGG-3′

To quantify viral reverse transcription products, we used Tat primers:

Tat forward: 5′- GAAGCATCCAGGAAGTCAGCC-3′,

Tat reverse: 5′- ACAAACTTGGCAATGAAAGCAACAC-3′

### Statistical analysis

Results are represented as mean ± SE for each bar graph. The significance between two groups was calculated using student's t-test. p<0.05 was considered significant.
